# 

**Published:** 1900-03

**Authors:** 


### CONTENTS:

### SELECTIONS.

Article I.—Dental Jurisprudence. F. F. Graves, D. D. S.......... 481
" II.—Another Life Elixir.—French Scientist's Alleged Discovery of Secret of Youth .......... 494
" III.—Useful Hints for the Laboratory. Arthur E. H. Lister .......... 496
" IV.—Method of Obtaining Perfect Metallic Dies for Gold or Aluminum Swaged Plates, Restoring all Undercuts as on Plaster Model. H. F. Grantvedt, D. D. S..... 498
" V.—Zinc Dies. Oliver Perry Wolfe, D. M. D .......... 500
" VI.—A Remarkable Reflex. Charles B. Kern, M. D.......... 502
" VII.—Clinical Demonstration of Dr. Bonwill's Methods of Practice. Reported by Geo. V. L. Brown, B. A., D. D. S., M. D., C. M.......... 504
" VIII.—The Most Remarkable Clinic in the History of Dentistry. Reported by Eugene S. Talbot, M. D., D. D. S.......... 509
" IX.—Vicarious Menstruation from the Gums. W. George Beers.......... 513

### COMMUNICATED.

The Wisconsin State Board of Dental Examiners.......... 514
The Kentucky State Dental Association.......... 519
The National Association of Dental Examiners.......... 519

### OBITUARY.

Dr. B. H. Catching.......... 520

### MONTHLY SUMMARY.

The Brunette People of Europe.......... 522

College Athletics.......... 523

Undercut Models.......... 525

Is the Enamel a Vital Tissue ?.......... 526

Death From Cutting a Wisdom Tooth.......... 526

Amalgam Fillings in Teeth.......... 527

Hot Water Drinking .......... 528

